# Dynamic prediction of survival using multivariate functional principal component analysis: A strict landmarking approach

**DOI:** 10.1177/09622802231224631

**Published:** 2024-01-09

**Authors:** Daniel Gomon, Hein Putter, Marta Fiocco, Mirko Signorelli

**Affiliations:** 1 Mathematical Institute, 4496 Leiden University, Leiden, the Netherlands; 2 Department of Biomedical Data Sciences, Leiden University Medical Centre, Leiden, the Netherlands

**Keywords:** Dynamic prediction, landmarking, survival, functional principal component analysis

## Abstract

Dynamically predicting patient survival probabilities using longitudinal measurements has become of great importance with routine data collection becoming more common. Many existing models utilize a multi-step landmarking approach for this problem, mostly due to its ease of use and versatility but unfortunately most fail to do so appropriately. In this article we make use of multivariate functional principal component analysis to summarize the available longitudinal information, and employ a Cox proportional hazards model for prediction. Additionally, we consider a centred functional principal component analysis procedure in an attempt to remove the natural variation incurred by the difference in age of the considered subjects. We formalize the difference between a ‘relaxed’ landmarking approach where only validation data is landmarked and a ‘strict’ landmarking approach where both the training and validation data are landmarked. We show that a relaxed landmarking approach fails to effectively use the information contained in the longitudinal outcomes, thereby producing substantially worse prediction accuracy than a strict landmarking approach.

## 1. Introduction

Routine collection of a wide array of repeatedly measured patient health outcomes is becoming more and more common, providing vital information about the current status of a patient’s health. Dynamically predicting future (adverse) events for individuals using their currently available information has therefore rapidly become of great interest. One of the main questions for this prediction problem is how to properly extract and use the information contained in the repeated measurements, especially if many variables are available for each subject.

Many different models already exist allowing for the prediction of survival probabilities from longitudinal data. Two commonly used methods are joint modelling (JM) and landmarking. JM of longitudinal outcomes and time-to-event data has risen in popularity as it can be used to simultaneously model the progression of longitudinal data and the survival outcome, allowing information between the two to be shared. A summary of recent developments and issues in JM approaches can be found in Hickey et al.^1^ There are a few downsides to JM: a (linear) model must be specified for the longitudinal outcomes, misspecifying the random effect structure in JM can lead to biased estimates and modelling many longitudinal outcomes quickly becomes computationally expensive. To deal with the last problem, Mauff et al.^2^ proposed a corrected two-stage approach to cut down computation time significantly, but to the best of the authors’ knowledge it is still not feasible to incorporate more than approximately ten longitudinal covariates in a JM model. The landmarking approach^3^ uses a Cox model with a *landmarked* data set containing only the values of the longitudinal variables until a so called landmark time. As an example, Van Houwelingen et al.^4^ have used this model to predict five-year failure-free survival after bone marrow transplantation. Nicolaie et al.^5^ further developed this model by incorporating competing risks and proposing a smoothed estimate over a collection of multiple landmarked data sets. As values of longitudinal covariates are not always known at the landmark time, an appropriate model (such as a mixed model) can be used to extrapolate these values in an approach that Ferrer et al.^6^ call ‘two-stage’ landmarking. The greatest advantage of landmarking methods is that they are very simple to implement: researchers can simply fit existing models on adjusted versions of the available data.

The traditional landmarking approaches described above utilize only the value of a longitudinal variable at the landmark time, ignoring information contained in the variable progression. A different approach can be taken where the longitudinal covariates and time-to-event data are modelled separately. This requires variable progression to first be described described using an appropriate model. For dynamic prediction this then entails a 3 -step procedure:

1. Landmark longitudinal and survival data.
2. Describe longitudinal trajectories using an appropriate model and extract summaries.
3. Supply above summaries to a survival model and use for future predictions.

In step 1 detailed above, a (representative) subset of the complete data should be used to train the model to reduce bias in parameter estimation. For dynamic prediction, the appropriate or ‘strict’ approach is to use only longitudinal data until the landmark time. In reality, many existing models do not perform landmarking strictly, instead training the model on all available data in what we call ‘relaxed’ landmarking. They usually employ either univariate/multivarite Functional Principal Component Analysis (u/mFPCA) or mixed modelling in step 2 to obtain summaries and either Cox regression or random survival forests (RSFs) in step 3 to link the summaries to the survival outcomes. A few examples using this relaxed approach include UFPCACox^7^ (uFPCA & Cox), MFPCACox^8^ (mFPCA & Cox), Functional RSFs^9,10^ (mFPCA & RSF) and pencal^11^ (Mixed models & Cox). On the other hand, Devaux et al.^12^ perform the landmarking procedure strictly using mixed models for the trajectories and consider both Cox models and RSF for the survival outcomes, also proposing to use a machine learning approach (superlearner) to combine the predictions from the considered models. Zhu et al.^13^ also used the strict landmarking approach, combining FPCA and Linear Transformation Models for the survival outcomes.

An assumption often made when analysing longitudinal data is that subjects are comparable at their entry time into the study. This assumption is implicitly present in FPCA models, where the mean progression of longitudinal variables is assumed to be the same for all subjects. Especially in an observational study this might not be the case, as participants will differ significantly in age at baseline. This gives rise to the hypothesis that there should be an effect of age on the longitudinal trajectories. As an example, brain mass has been shown to increase and decrease throughout the human lifespan.^14^ Seeing as age can be included in the baseline predictors, we would therefore like to eliminate the natural variation in the longitudinal trajectories caused by the age disparity between subjects before performing further analyses. We propose an age-based centred (ABC) mFPCA procedure, where the mean value of the longitudinal outcomes is assumed to depend on the age of the single subject. A comparable idea called generalized landmarking analysis has recently been explored by Yao et al.^15^

The main focus of this article will be to examine whether we can improve the predictions of the MFPCCox model proposed by Li et al.^8^ by using a strict landmarking approach and eliminating the natural variation in the longitudinal variables by using an ABC mFPCA procedure.

This paper is structured as follows. In Section 2 we introduce the notation and discuss the proposed methods. Section 2.6 provides a brief overview of the considered methods. We evaluate the proposed models in Section 3 by means of a simulation study. In Section 4 we apply all different methods on an observational study on Alzheimer’s disease (AD). The article ends with a discussion in Section 5.

## 2. Methods

In this section, we describe a three-step approach to dynamically predict survival probabilities.

### 2.1. Notation

Consider a study with patients i=1,…,n and patient specific visit times $t_{ij}$ with $j=1,\ldots ,m_{i}$ , where $t_{ij}$ denotes the time from the baseline visit so that $t_{i1}=0$ for each i . We either observe the true time to event $T_{i}$ or an independent right censoring time $C_{i}$ for each patient. The observed event time is then $T_{i}^{*}=\min\{T_{i},C_{i}\}$ . Let $\delta_{i}\in \{0,1\}$ be a censoring indicator, with $\delta_{i}=1$ indicating that the true event time has been observed.

At the first patient visit, P baseline biomarkers $Z_{i}=(Z_{i1},\ldots ,Z_{iP}{)}^{⊤}$ are observed with $a_{i}\in Z_{i}$ denoting the age of a patient at baseline. At each visit time j , a vector of Q covariates $Y_{ij}=(Y_{ij}^{\left(1\right)},\ldots ,Y_{ij}^{\left(Q\right)}{)}^{⊤}$ is measured. Denote by $Y_{i}^{\left(q\right)}=(Y_{i1}^{\left(q\right)},\ldots ,Y_{im_{i}}^{\left(q\right)}{)}^{⊤}$ with q=1,…,Q the full information on a single longitudinal covariate of patient i . The matrix $Y_{i}=(Y_{i}^{\left(1\right)},\ldots ,Y_{i}^{\left(Q\right)}{)}^{⊤}$ contains all available longitudinal information for a single individual i . To make the dependence on time explicit we define $Y_{i}^{\left(q\right)}(t_{ij}):=Y_{ij}^{\left(q\right)}$ . To summarize, we observe Q longitudinal outcomes (superscript (q) ) for n patients (subscript i ) at $m_{i}$ times (subscript j ) since their entry into the study.

Let $t_{LM}>0$ be the time at which we wish to make predictions for the future, called the landmark time. The ultimate goal is to predict the probability of survival for a patient still alive at the landmark time, using all information available until that point in time. To formalize this, we introduce the history of the longitudinal variables until time t for subject i as $Y_{i,t}=\{(Y_{i1}^{\left(q\right)},\ldots ,Y_{ik_{i}}^{\left(q\right)}{)}^{⊤};k_{i}\in \{1,\ldots ,m_{i}\},t_{ik_{i}}\leq t,q=1,\ldots ,Q\}$ with $t_{ik_{i}}$ the last patient specific visit time at or before landmark time. In other words, we wish to find:

$$\pi_{i}(t|t_{LM})=P(T_{i}>t|T_{i}>t_{LM},Z_{i},Y_{i,t_{\mathrm{LM}}})$$

with $t>t_{\mathrm{LM}}$ .

In (dynamic) prediction modelling, the data is usually separated into a training and test data. The training data is used to build a prediction model and the performance of the model is then evaluated on the test data. A common approach is to use (repeated) k -fold cross-validation, where all data is randomly split into k folds of equal size, with k−1 folds used for training and one for testing.

### 2.2. Step 1: Landmarking

The first step in our method will be to landmark the data, removing information that can skew predictions for future patients.

#### 2.2.1. Strict landmarking

We consider a ‘strict landmarking’ approach for dynamic prediction as proposed by Van Houwelingen.^3^ We landmark both the training and the testing data set by removing all patients with an observed event before a landmark time $t_{\mathrm{LM}}$ , leaving only patients with $T_{i}^{*}>t_{LM}$ . Heuristically, patients with an event before the landmark time are not representative of the prediction problem as we are only trying to predict survival probabilities for patients who have not yet experienced an event at landmark time.

We only train our model on the longitudinal trajectories of the remaining patients until landmark time. In other words, we truncate the data $Y_{i}$ at time $t_{\mathrm{LM}}$ such that $t_{im_{i}}\leq t_{\mathrm{LM}}$ for all patients.

#### 2.2.2. Relaxed landmarking

A popular approach is to use what we call ‘relaxed landmarking’. In this landmarking approach, the training model uses the complete information of all individuals in the training set. For the prediction set, only the observations up until landmark time are used. This means that more information is used to build the model than is available at the time of prediction. This can lead to a biased model, as patients that have already experienced an event at the landmark time are not likely to follow the same longitudinal patterns as patients without an event at the landmark time. The relaxed landmarking approach has already been used before on the Alzheimer Disease Neuroimaging Initiative (ADNI) data set considered in Section 4.1.^8,9,16^

### 2.3. Step 2: Functional principal component analysis

For each q=1,…,Q consider a square integrable process $X^{\left(q\right)}:T\to R$ , with $X_{i}^{\left(q\right)}(t_{ij})$ a realization from this process at visit time $t_{ij}$ . Assume that the observed longitudinal covariates are the sum of a realization from such a process (which we will call the underlying process) and a measurement error, so that $Y_{i}^{\left(q\right)}(t_{ij})=X_{i}^{\left(q\right)}(t_{ij})+\epsilon_{ij}^{\left(q\right)}$ with $\epsilon_{ij}^{\left(q\right)}∼N(0,\sigma^{2})$ iid. The Q available longitudinal covariates can be described by continuous processes and summarized using u/mFPCA. FPCA methods aim to project longitudinal data on an ‘optimal’ choice of basis, thereby reducing the dimension and allowing for summary statistics to be extracted.^17,18^ The notation in this section was inspired by Happ and Greven.^19^

#### 2.3.1. uFPCA

For each q=1,…,Q let $X^{\left(q\right)}:T\to R$ be defined on a domain T with mean $E[X^{\left(q\right)}(t)]=\mu^{\left(q\right)}(t)$ and covariance function $C^{\left(q\right)}(s,t)=\mathrm{Cov}(X^{\left(q\right)}(s),X^{\left(q\right)}(t))$ . Mercer’s theorem states that the covariance function can be decomposed as follows:

$$C^{\left(q\right)}(s,t)=\sum_{m=1}^{\infty }\lambda_{m}^{\left(q\right)}\phi_{m}^{\left(q\right)}(s)\phi_{m}^{\left(q\right)}(t)$$

where $\phi_{m}^{\left(q\right)}$ is a set of orthonormal eigenfunctions and $\lambda_{1}^{\left(q\right)}\geq \lambda_{2}^{\left(q\right)}\geq \ldots ,\geq 0$ are eigenvalues of the associated autocovariance operator $(Af{)}^{\left(q\right)}(t)=\int_{T}f(s)C^{\left(q\right)}(s,t)ds$ . By the Karhuhen-Loève Theorem we can decompose the process:

(1) $$X^{\left(q\right)}(t)=\mu^{\left(q\right)}(t)+\sum_{m=1}^{\infty }\xi_{m}^{\left(q\right)}\phi_{m}^{\left(q\right)}(t)$$

where $\xi_{m}^{\left(q\right)}$ is a set of uncorrelated random variables with $E[\xi_{m}^{\left(q\right)}]=0$ and $\mathrm{Var}(\xi_{m}^{\left(q\right)})=\lambda_{m}^{\left(q\right)}$ . The $\xi_{m}^{\left(q\right)}$ are called the principal components or scores. Note that the scores can be correlated across different covariates. They can be recovered as:

(2) $$\xi_{m}^{\left(q\right)}=\int_{T}\left(X^{\left(q\right)}(s)-\mu^{\left(q\right)}(s)\right)\phi_{m}^{\left(q\right)}(s)ds$$

The process $X^{\left(q\right)}(t)$ can be expanded in the same way in any orthonormal basis, but the basis $\left\{\phi_{m}^{\left(q\right)},m\geq 1\right\}$ maximizes the variance $\lambda_{m}^{\left(q\right)}$ of the principal components, thereby explaining the largest amount of variation compared to other choices of basis (see Chapter 8.2 of Ramsay and Silverman^17^).

Consider the values of the q -th observed biomarker for each patient $Y_{i}^{\left(q\right)}(t_{ij})=X_{i}^{\left(q\right)}(t_{ij})+\epsilon_{ij}^{\left(q\right)}$ as a noisy realization from the process $X^{\left(q\right)}(t)$ . uFPCA allows to summarize the process for each patient by projecting onto the eigenfunctions $\phi_{m}^{\left(q\right)}$ and obtaining individual scores $\xi_{i,m}^{\left(q\right)}$ . To obtain these eigenfunctions and scores we first need to determine an estimate for the mean function $\mu^{\left(q\right)}(t)$ and the covariance function $C^{\left(q\right)}(s,t)$ of the underlying process. For this, we use the R package MFPCA.^20^ MFPCA estimates a smooth mean function ${\hat{\mu }}^{\left(q\right)}(t)$ by fitting a thin plate regression spline on the pooled data of all training observations using the R package mgcv.^21^ Thin plate regression splines minimize the ‘wiggliness’ of the estimated smoother, creating ‘visually smooth’ estimates for the mean function (Chapter 5.5 of Wood^21^). Similarly, the covariance function $C^{\left(q\right)}(s,t)$ is estimated by fitting a tensor product smooth on the pooled patient sample covariances:

$$G_{i}^{\left(q\right)}(t_{ij},t_{ij^{\prime }})=\left(Y_{ij}^{\left(q\right)}(t_{ij})-{\hat{\mu }}^{\left(q\right)}(t_{ij})\right)\left(Y_{ij^{\prime }}^{\left(q\right)}(t_{ij^{\prime }})-{\hat{\mu }}^{\left(q\right)}(t_{ij^{\prime }})\right)$$

ignoring the diagonal as there we get biased estimates: $E[G_{i}^{\left(q\right)}(t_{ij},t_{ij^{\prime }})]=\mathrm{Cov}(X^{\left(q\right)}(t_{ij}),X^{\left(q\right)}(t_{ij^{\prime }}))+\sigma^{2}1_{jj^{\prime }}$ . This yields an estimator for the covariance function ${\hat{C}}^{\left(q\right)}(s,t)$ . The diagonals are used to estimate the observation error variance $\hat{\sigma^{2}}$ . We then estimate the eigenvectors ${\hat{\phi }}_{im}^{\left(q\right)}=({\hat{\phi }}_{m}^{\left(q\right)}(t_{i1}),\ldots ,{\hat{\phi }}_{m}^{\left(q\right)}(t_{im_{i}}){)}^{⊤}$ by a spectral decomposition of the matrix ${\hat{\Sigma }}_{X_{i}^{\left(q\right)}}={\left({\hat{C}}^{\left(q\right)}(t_{ij},t_{ij^{\prime }})\right)}_{jj^{\prime }}$ with $j,j^{\prime }\in \{1,\ldots ,m_{i}\}$ . The scores are estimated using principal analysis by conditional expectation (PACE)^22^ instead of numerically calculating the integral in Equation (2):

(3) $${\hat{\xi }}_{im}^{\left(q\right)}=E[\xi_{im}^{\left(q\right)}|Y_{i}^{\left(q\right)}]={\hat{\lambda }}_{m}^{\left(q\right)}{{\hat{\phi }}_{im}^{\left(q\right)}}^{⊤}{\hat{\Sigma }}_{Y_{i}^{\left(q\right)}}^{-1}\left(Y_{i}^{\left(q\right)}-{\hat{\mu }}^{\left(q\right)}\right)$$

where ${\hat{\Sigma }}_{Y_{i}^{\left(q\right)}}={\hat{\Sigma }}_{X_{i}^{\left(q\right)}}+\hat{\sigma^{2}}{\text{I}}_{m_{i}}$ . The processes are truncated using a suitable number of principal components so that $X_{i}^{\left(q\right)}(t)\approx \mu^{\left(q\right)}(t)+\sum_{m=1}^{M^{\left(q\right)}}\xi_{m}^{\left(q\right)}\phi_{m}^{\left(q\right)}(t)$ . Usually $M^{\left(q\right)}$ is chosen so that the truncated components explain a required proportion of the total variance: $\frac{\sum_{m=1}^{M^{\left(q\right)}}\lambda_{m}}{\sum_{m=1}^{\infty }\lambda_{m}}>{\mathrm{PVE}}^{\left(q\right)}$ , where PVE is the Proportion of total Variance Explained.

#### 2.3.2. mFPCA

Instead of decomposing each of the q=1,…Q longitudinal trajectories into univariate eigenfunctions ${\hat{\phi }}_{m}^{\left(q\right)}$ and scores ${\hat{\xi }}_{im}^{\left(q\right)}$ separately, it can be preferable to consider the multivariate process $X_{i}(t)=(X_{i}^{\left(1\right)}(t),\ldots ,X_{i}^{\left(Q\right)}(t){)}^{⊤}$ instead as this allows to summarize all longitudinal variables in a smaller number of principal components. The multivariate versions of Mercer’s theorem and the Karhuhen-Loève theorem allow us to decompose the individual trajectories in a similar manner as before:

(4) $$X_{i}(t)=\mu (t)+\sum_{m=1}^{\infty }\rho_{im}\psi_{m}(t)\approx \mu (t)+\sum_{m=1}^{M}\rho_{im}\psi_{m}(t)$$

with multivariate mean function $\mu (t)=(\mu^{\left(1\right)}(t),\ldots ,\mu^{\left(Q\right)}(t){)}^{⊤}$ , multivariate eigenfunctions $\psi_{m}:T\to R^{Q}$ and principal components/scores $\rho_{im}\in R$ . The truncation parameter M can then be chosen such that $M\geq \sum_{q=1}^{Q}M^{\left(q\right)}$ , where $M^{\left(q\right)}$ is chosen such that a sufficient proportion of variance is explained in the associated univariate process. In summary, mFPCA describes the collection of all longitudinal variables using a multi-dimensional framework.

Happ and Greven^19^ show that there is a one-to-one correspondence between the univariate and multivariate decomposition(s). Using this correspondence it is possible to obtain the multivariate scores and eigenfunctions from Q univariate decompositions. This allows to represent a single patient by M uncorrelated scores $\rho_{im}$ , whereas using uFPCA we would require $\sum_{q=1}^{Q}M^{\left(q\right)}$ (possibly correlated) scores $\xi_{im}^{\left(q\right)}$ . The R^23^ package MFPCA^20^ allows to determine both the univariate and multivariate decomposition(s) of the observed processes.

By fitting the model on the training data we obtain eigenfunctions and eigenvalues, as well as patient specific scores. To determine scores for new patients in the test data, we can follow the same procedure, starting with equation (3). Note that except for $Y_{i}^{\left(q\right)}$ , all components of equation (3) are estimated from the training set and are either fixed or only depend on the time of observations. Univariate scores for test data can therefore be predicted quite easily. To obtain multivariate scores, we once again exploit the one-to-one correspondence in Happ and Greven.^19^

#### 2.3.3. Age-based centering

In equations (1) and (4) both the mean functions and eigenfunctions are modelled on the time-on-study scale. However, for most covariates of interest modelling their dynamic evolution as a function of time-on-study may be unrealistic, as one would expect the value of the longitudinal covariates to depend on the age of the patient, rather than on the time since their entry into the study. For example, brain mass is unlikely to depend on the time spent in a study but has been shown to depend on the age of the subject.^14^ For this reason, we consider an alternative modelling approach where the mean of the longitudinal processes $X_{i}(t)$ depends on the age of the patient:

(5) $$X_{i}(t)\approx \mu (t,a_{i})+\sum_{m=1}^{M}\rho_{im}\psi_{m}(t)$$

where $a_{i}$ is the age of patient i at baseline and:

(6) $$\mu (t,a_{i})={\left(\mu^{\left(1\right)}(a_{i}+t),\ldots ,\mu^{\left(Q\right)}(a_{i}+t)\right)}^{⊤}$$

In this way, the mean function is only determined by the age of the patient at the observation time, while the variations between patients are observed in the study time. We call this procedure age-based centring and consider ABC mFPCA to be the mFPCA procedure under this assumption.

To obtain an estimate $\hat{\mu }(t,a_{i})$ of the mean function, we pool the translated observations to obtain $M_{ABC}:=\{(t_{ij}+a_{i},Y_{i}^{\left(q\right)}(t_{ij}));j=1,\ldots ,m_{i}{\}}_{i=1,\ldots ,n}$ . We then produce a smooth estimate of the mean function by fitting a thin plate regression spline on this pooled data using the mgcv^21^ package.

#### 2.3.4. Strict and relaxed landmarking: mFPCA estimation

When using relaxed landmarking, the uFPCA models are built on the complete trajectories of patients in the training set. This means that there is usually plenty of information to train the model, even when there are a lot of missing observations. When strict landmarking is applied, the training models are only built on the trajectories until landmark time. Besides this, patients with an event time before the landmark time are not considered, meaning that the prediction model is trained on a much smaller set of data. Combined with missingness in the data, this can lead to difficulties in the estimation of the mean functions $\mu^{\left(q\right)}(t)$ and covariance functions $C^{\left(q\right)}(s,t)$ . In practice, this means that fewer basis functions are used in the smoothing steps of thin plate spline regression with strict landmarking.

Another issue arises for data with a large proportion of missing data. The scores for both training and test data are calculated using PACE (see equation (3)). For each patient this requires the calculation of ${\hat{\Sigma }}_{Y_{i}^{\left(q\right)}}^{-1}$ . When $\hat{\sigma^{2}}$ is estimated to be zero (due to a lack of training data) and the patient in question has fewer observations before landmark time than the required number of principal components, ${\hat{\Sigma }}_{Y_{i}^{\left(q\right)}}$ cannot be inverted. In this situation, we calculate the maximum possible number of principal components (which is equal to their number of observations before landmark time) and set the rest of the components to zero. Software to perform this estimation task as well as an ABC mFPCA procedure can be found in an adjusted MFPCA package at github.com/d-gomon/MFPCA.

### 2.4. Step 3: Dynamic prediction of survival

In step 2 we estimated patient specific scores ${\hat{\rho }}_{i}=({\hat{\rho }}_{i1},\ldots ,{\hat{\rho }}_{iM}{)}^{⊤}$ , summarising their longitudinal trajectories. We would now like to dynamically predict the probability of uncensored survival for a patient i still alive at landmark time $t_{LM}$ :

(7) $$\pi_{i}(t|t_{LM})=P(T_{i}>t|T_{i}>t_{LM},Z_{i},{\hat{\rho }}_{i})$$

We model the underlying hazard in the training data by using the Cox proportional hazards model:

(8) $$h(t|Z_{i},{\hat{\rho }}_{i})=h_{0}(t)e^{\beta^{⊤}Z_{i}+\gamma^{⊤}{\hat{\rho }}_{i}}$$

where the coefficients vectors $\hat{\beta }$ and $\hat{\gamma }$ are estimated by using Efron’s partial likelihood^24^ and the baseline hazard $\hat{h_{0}}(t)$ using the Breslow estimator^25^ employed in the R package survival.^26^ We can also choose to fit a regularized Cox model using glmnet^27^ which estimates coefficients using coordinate descent. We choose to only regularize the γ coefficients associated with the longitudinal scores. The predicted survival probabilities for a new patient i are:

$${\hat{\pi }}_{i}(t|t_{LM})={\left(\frac{\hat{S_{0}}(t)}{\hat{S_{0}}(t_{\mathrm{LM}})}\right)}^{\exp\;({\hat{\beta }}^{⊤}Z_{i}+{\hat{\gamma }}^{⊤}{\hat{\rho }}_{i})}$$

where $\hat{S_{0}}(t)=\exp\;(-\int_{0}^{t}\hat{h_{0}}(s)ds)$ . Notice that when strict landmarking is performed $\hat{S_{0}}(t_{LM})=1$ .

#### 2.4.1. Regularization

When the number of longitudinal covariates is large, the number of scores M can also grow quickly making the problem high-dimensional. The scores in the mFPCA decomposition describe how closely an individual patient follows the trajectory of the associated eigenfunction or ‘trend’. We do not expect all trends to be predictive for the survival of a specific patient in the future. Regularized Cox regression^28^ can be used to solve these problems. We use the R package glmnet,^27^ which can fit Ridge, LASSO and elastic-net Cox regression models. In this article, we limit ourselves to LASSO regression as this allows us to shrink the coefficients in the Cox model and select only the trends which are important for prediction.

### 2.5. Model validation

We evaluate the accuracy of the predicted survival probabilities ${\hat{\pi }}_{i}(t|t_{LM})$ using time dependent AUC (tdAUC) and Brier score (BS). The former is defined as the area under the time-dependent ROC curve^29^ and measures the discrimination potential of the model at the prediction time t as a trade-off between sensitivity and specificity. This indicates how well we can predict whether someone will survive past the prediction time or not, with a value of 1 indicating perfect discrimination, whereas 0.5 indicates random guessing. The BS^30^ measures the squared distance between the predicted survival probability and the status indicator at the prediction time. A value of 0 indicates that we predict the survival probabilities perfectly, while large values indicate that the predictions are biased. We use the R package riskRegression^31^ to calculate these validation scores.

We employ repeated cross-validation to obtain unbiased estimates of predictive performance (as measured by tdAUC and BS) by repeatedly splitting all available data randomly into k−1 training folds to estimate the model and 1 validation fold to evaluate the performance. In case of strict landmarking, all data is first landmarked at the required landmark time. When relaxed landmarking is performed, we first subdivide the data into folds and afterwards landmark only the prediction fold at the landmark time. This is a consequence of using full trajectories of all patients for training in the relaxed landmarking method. The whole process is repeated l times for both methods after which the performance measures are averaged over folds and repeats. By repeating the cross validation procedure multiple times we can obtain a more robust estimate for the performance measures.

### 2.6. Summary

There are three choices to be made in the methods discussed above:

- Strict/Relaxed Landmarking.
- Standard/ABC mFPCA.
- Standard/Regularized Cox regression.

**Figure 1.**
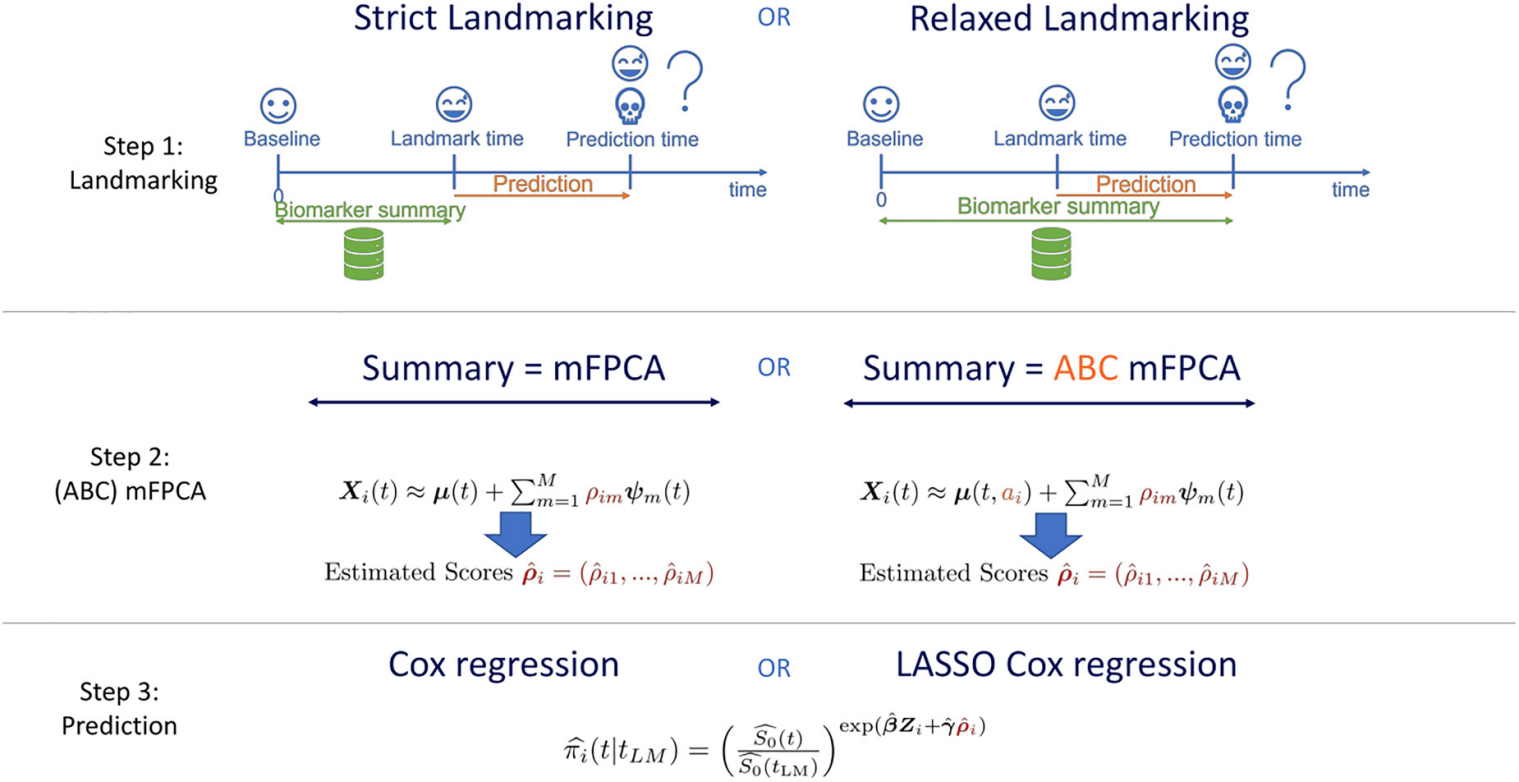
Graphical summary of the methods proposed in Section 2. See also Section 2.6.

The methods are also graphically summarized in Figure 1. The model with relaxed landmarking, the standard mFPCA procedure and standard Cox regression is the MFPCCox framework proposed by Li and Luo.^8^ We will consider MFPCCox as a reference method and evaluate the effect of the considered additions by comparing the performance between methods in a simulation study as well as on a real data set.

## 3. Simulation study

In this section, we perform a simulation study to evaluate the impact on prediction accuracy of the following choices in 3 -step mFPCA models: (a) strict/relaxed landmarking; (b) wrongly assuming age-at-observation/time-on-study mean functions for the longitudinal processes.

### 3.1. Data generation

We consider a study where n=1600 subjects are followed up at quarter-yearly intervals over the time span of 15 years so that $t_{ij}\in \{0,0.25,\ldots ,15\}$ for all i ( i=1,…,1600 ) and j ( j=1,…,61 ). Subject’s age at the baseline measurement is uniformly distributed between 40 and 90 years old, so that all age groups are well represented in the data. For each subject i we observe Q=3 longitudinal variables. Since we are primarily interested in the performance of the mFPCA methods in recovering longitudinal patterns, we do not include baseline variables in the simulation study ( P=0 ). We generate the underlying longitudinal processes $X_{i}(t)$ as in (4) (**time-on-study data**) or (5) (**age-at-observation data**) with M=6 (two ‘trends’ per variable). The scores $\rho_{im}$ are generated from normal distributions with decreasing variance $\nu_{m}\in \{1,\frac{5}{6},\frac{2}{3},0.5,\frac{1}{3},\frac{1}{6}\}$ . The eigenfunctions $\psi_{m}(t)$ are generated using the ‘split’ method described in Section 2 of the online supplement to Happ and Greven^19^ using the first six Fourier basis functions. The mean functions are given by:

(9) $$\begin{array}{rl} \mu (t)=\left(\begin{array}{c} 20-(\frac{t}{3}-3{)}^{2}\\ \log\;(t+1)\\ \exp\;\left(-\frac{t-10}{5}\right)+5 \end{array}\right); & \mu (t,a_{i})=\left(\begin{array}{c} 20-(\frac{a_{i}+t-73}{5}{)}^{2}\\ \log\;\left((a_{i}+t-40{)}^{6}\right)-10\\ \exp\;\left(-\frac{a_{i}+t-40}{20}\right)+5 \end{array}\right) \end{array}$$

**Figure 2.**
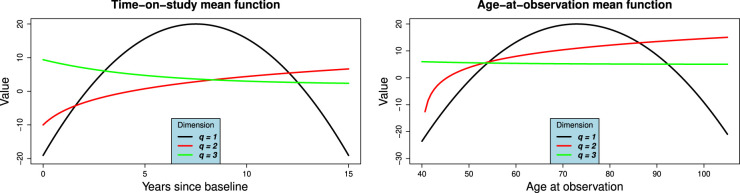
(a) Time-on-study and (b) Age-at-observation mean functions for simulation study. The functions correspond to equation (9).

The time-on-study and age-at-observation mean functions have similar progressions, but on a different time scale. The mean functions are displayed in Figure 2. The observed longitudinal variables are obtained as $Y_{i}^{\left(q\right)}(t_{ij})=X_{i}^{\left(q\right)}(t_{ij})+\epsilon_{ij}^{\left(q\right)}$ , with $\epsilon_{ij}^{\left(q\right)}∼N(0,0.1^{2})$ independent observation errors.

Survival times are generated by applying the inverse transformation method^32^ as follows. Consider the subject-specific hazard function:

(10) $$h_{i}(t)=h_{0}(t)\exp\;\left(\sum_{q=1}^{3}\alpha^{\left(q\right)}\eta_{i}^{\left(q\right)}(t)\right)$$

with $\eta_{i}^{\left(q\right)}(t)=\sum_{m=1}^{6}\rho_{im}\psi_{m}^{\left(q\right)}(t)$ and α=(1,−1,2) and a Weibull baseline hazard with shape and scale parameters equal to 3 and 8.4 respectively. Using these parameters we obtain an approximately symmetric distribution with a mean survival time of around 7.5 years (halfway through the study period). We draw a survival probability $S_{i}(T_{i})$ for each subject from the standard uniform distribution and then obtain survival times by numerically solving for $T_{i}$ :

$$S_{i}(T_{i})=\exp\;\left(-\int_{0}^{T_{i}}h_{i}(t)dt\right)$$

Finally, right censoring times $C_{i}$ are generated using an exponential distribution with rate λ ; the survival time is censored if $C_{i}<T_{i}$ . To evaluate the effect of censoring on the predictive performance we consider three different censoring rates: λ=1.48 (light censoring, ≈20% of observations censored), λ=2.16 (median censoring, ≈40% of observations censored) and λ=2.88 (heavy censoring, ≈60% of observations censored). This results in the following 6 simulation scenarios:

Scenario1

Time-on-study data and light censoring

Scenario2

Time-on-study data and median censoring

Scenario3

Time-on-study data and heavy censoring

Scenario4

Age-at-observation data and light censoring

Scenario5

Age-at-observation data and median censoring

Scenario6

Age-at-observation data and heavy censoring

Note that the hazard used to simulate survival outcomes (equation (10)) is not the same as the one used by the models (equation (8)). If we were to simulate survival times from the hazard in equation (8) we would favour relaxed landmarking approaches as the scores that influence the survival times can only accurately be determined from the full follow-up duration in that case. We therefore take a different approach, where the hazard depends on the current value of the longitudinal trajectories for each patient. With this procedure neither the strict nor relaxed landmarked model is correctly specified. We believe however that this simulation procedure is more realistic, as it seems more likely that the progression of biomarkers is predictive for the occurrence of an event as opposed to some underlying latent factor that influences the biomarkers trajectories. An important consideration in this simulation study is that the age of a subject does not influence their survival directly, but only through how well the true scores can be recovered from the generated data after substracting the estimated mean function.

Having simulated longitudinal covariates (both on the time-on-study and age-at-observation scale) and survival times in each scenario, we now apply the models discussed in Section 2. We do not consider regularization in the simulation study, as we are working in a low-dimensional setting and the true scores are uncorrelated. We choose $M^{\left(q\right)}$ such that ${\mathrm{PVE}}^{\left(q\right)}\geq 0.95$ for all q=1,2,3 . At each validation time point we can calculate the mean squared error (MSE) between the predicted probabilities and true probabilities for the $n^{\prime }$ people in the prediction set:

$$\mathrm{MSE}(t)=\frac{\sum_{i=1}^{n^{\prime }}{\left(\pi_{i}(t|t_{LM})-{\hat{\pi }}_{i}(t|t_{LM})\right)}^{2}}{n^{\prime }}$$

Since the true underlying hazard function $h_{i}(t)$ is known the true probability of event-free survival $\pi_{i}(t|t_{LM})$ at time t can be computed. We also compute tdAUC and BS using the true probabilities, indicating the best prediction performance that can be achieved.

The main goals of our simulation study are to evaluate the effect on prediction accuracy of three components in the proposed methods. First of all, we are interested in what happens when the data-generating mechanism has been misspecified in the model: What happens to ABC models in a time-on-study scenario and what happens when not performing ABC in an age-at-observation scenario? For this reason, we generate data in both mechanisms. Secondly, we want to explore whether strict landmarking can outperform relaxed landmarking or vice versa? Which one yields the best predictions and does this depend on the landmark time? To examine this, we consider three different landmark times at three, six and nine years after the start of the study. With the employed survival generation mechanism, most people will still be at risk at the first landmark time, approximately half will have had an event at six years and most people will have failed by the last landmark time. Finally, we are curious to see how censoring patterns affect the predictive performance of the models and whether it influences the previous two points. We therefore consider the three different censoring patterns (light/median/heavy).

### 3.2. Results

**Figure 3.**
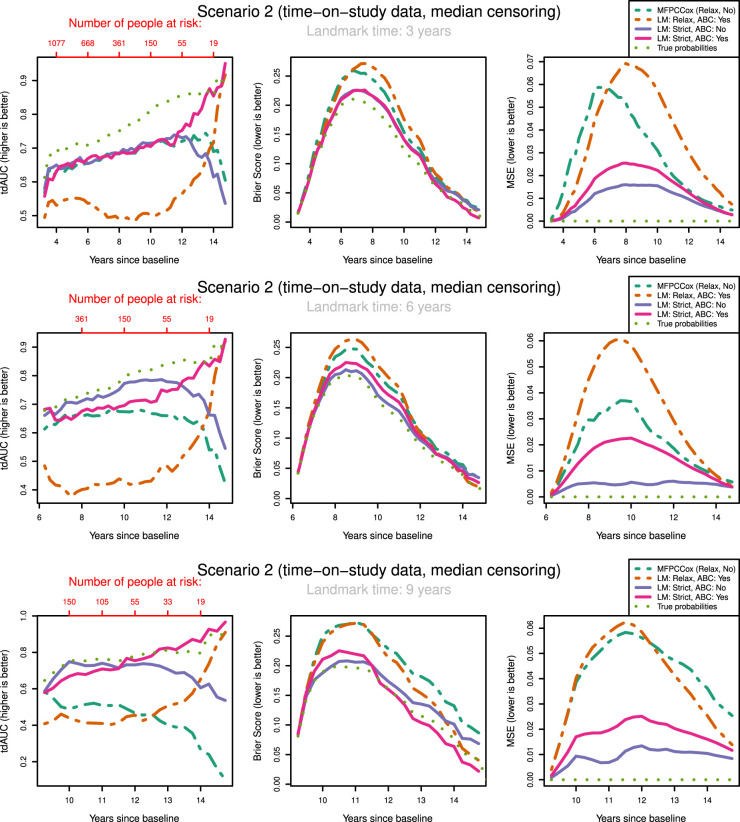
tdAUC, Brier Score and MSE in the second scenario (time-on-study data, median censoring) for the considered methods over landmark times at 3,6 and 9 years after baseline. Dashed lines: Relaxed landmarked methods. Solid lines: Strictly landmarked methods. Dotted lines: True probabilities. MFPCCox^8^ (LM: Relax, LASSO: No, ABC:No) used as reference method. Number of people at risk at evaluation times displayed in red. (a) Landmark time: 3 years; (b) Landmark time: 6 years; (c) Landmark time: 9 years. tdAUC: time-dependent AUC; MSE: mean squared error; LM: Landmark method; ABC: age-based centred;

**Figure 4.**
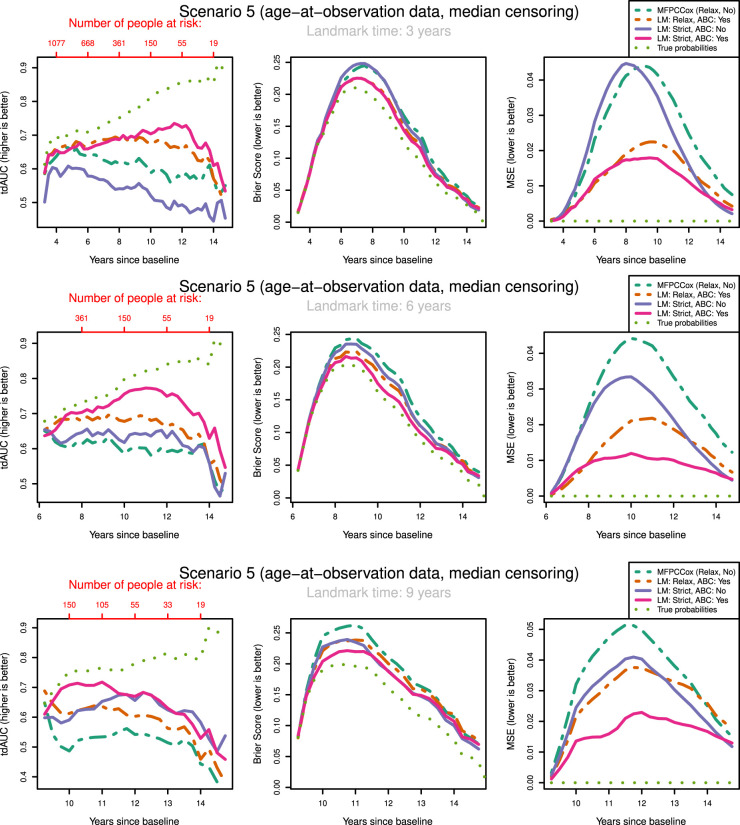
tdAUC, Brier Score and MSE in the fifth scenario (age-at-observation data, median censoring) for the considered methods over landmark times at 3,6 and 9 years after baseline. Dashed lines: Relaxed landmarked methods. Solid lines: Strictly landmarked methods. Dotted lines: True probabilities. MFPCCox^8^ (LM: Relax, LASSO: No, ABC:No) used as reference method. Number of people at risk at evaluation times displayed in red. (a) Landmark time: 3 years; (b) Landmark time: 6 years; (c) Landmark time: 9 years. ABC: age-based centred; LM: Landmark method; tdAUC: time dependent AUC; MSE: mean squared error.

To keep the results clear, we only show the results for median censoring (scenarios 2 and 5 ) in Figures 3 and 4. The results for light and heavy censoring can be found in Supplemental Figures 1−4 . To compare methods on their predictive performance we mainly focus on MSE, as for individual dynamic prediction we are mostly concerned with how well a model recovers the true probability of failure for a subject. The BS can be seen as an ‘estimate’ of the MSE using the observed outcomes when the true failure probabilities are not known. We can therefore also determine whether the BS can be reliably used to compare the methods in a real-life application. Finally, the tdAUC is a measure of the discriminatory potential of the methods. Although interesting in theory, it is not a vital measure for individual dynamic prediction.

The most notable result is that in both scenarios and over all landmark times the strictly landmarked correctly specified model performs best in MSE. Let us compare the MSE of the ABC methods between strict and relaxed landmarking in Scenario 5 (Figure 4). We find that at early landmark times, the strict and relaxed landmarked methods perform similarly. Notably, at landmark times of 3 and 6 years the relaxed method has a slightly better MSE for short prediction horizons. As we increase the landmark time strict landmarking considerably outperforms relaxed landmarking. This is in line with what we expect, as at early landmark times both methods will build the model on approximately the same set of patients. The longitudinal information used to build the model differs between the methods however, explaining the difference in predictive power. As the landmark time increases, so does the difference between the training sets for the two models. A similar trend can also be seen in scenarios 4 and 6 . On the other hand, this trend is not as pronounced in the time-on-study scenarios ( 1−3 ). Remember that strict landmarking methods determine the mean function $\mu (t,a_{i})$ or μ(t) using only the information available until landmarking time for each subject that is still alive. In a time-on-study scenario more data is available to estimate this mean function as each subject contributes to the overall estimate independent of their age, thereby mitigating this disadvantage of strict methods with respect to relaxed methods.

We might expect that strict landmarking methods will perform worse at later landmark times as they are using less data to build the models, seeing as most subjects will have had an event at later times and therefore be removed from the training set. This reverse is the case, with relaxed landmarking performing worse as the landmark time increases. This is likely due to relaxed methods using a very unsuitable training set: using data from people who have already had an event and using future observations to predict past ones.

Surprisingly, we find that Strict ABC landmarking can perform extremely well in the time-on-study scenarios when considering tdAUC and BS. At some prediction time points, the predictive performance of this model can even exceed that of the perfect prediction model, see for example Figure 3(c). Additionally, the Relaxed ABC method can also perform very well at later time points. Although looking at the Brier score we would conclude that both these methods are performing better than the correctly specified strict landmarking, we can see that in MSE they are performing significantly worse. Upon examining the intermediary results of these models, we found that the misspecified relaxed/strict models were estimating scores with extremely high variances which were not found to have predictive power in the resulting Cox model. Further inspecting the predicted probabilities of these models, we found that these were indeed very suitable to discriminate between subjects at later time points, but recovered the true probabilities very poorly. Note that this only ‘improves’ their predictive capabilities when very few people are at risk. When looking only at tdAUC and BS measures however, this can lead us to believe that they are performing very well. This can be a problem for evaluating the prediction of the models on real-life data.

The question arises whether correctly specifying the landmarking approach or the mean function is most important. We can see in Figure 3 that strict landmarking approaches perform best (in MSE), with correctly specified ABC performing best. The relaxed methods display significantly worse MSE over all landmark times and all censoring patterns (see also Supplemental Figures 1 and 2 ). The reason for this is likely that ABC methods are more flexible in modelling the mean function, negating the negative effect of misspecifying the data generation mechanism slightly. On the other hand, in the age-at-observation scenarios correctly specifying the data generation mechanism is more important for recovering proper survival estimates, especially at early landmark times. At later landmark times, strictly landmarking becomes vital again.

In our discussions above it seems that there are multiple biases working against each other, making it difficult to pinpoint what exactly causes one method to perform better or worse than the other. In general, we can conclude that relaxed landmarking introduces an estimation bias into the mFPCA procedure due to using future observations and an unsuitable training set. We find this bias to be largest when the training sets between the methods differ most, which in our simulation study is at later landmark times as then most subjects will already have experienced an event. On the other hand, there is also the bias of misspecifying the data generation mechanism. This can result in worse estimates for the survival probabilities, but has less of an impact in the time-on-study scenarios.

Let us examine how censoring influences the predictive potential of the models. In both the time-on-study and age-at-observation scenarios, the degree of censoring does not influence the performance between strict landmarking methods in MSE. For relaxed landmarking models, it is unclear what the influence of censoring is on the performance. For light and median censoring correctly specified relaxed landmarking performs better than incorrectly specified relaxed landmarking, especially at earlier landmark times. At later landmark times, both have comparable performance in MSE. In heavy censoring scenarios, incorrectly specified relaxed landmarking can perform better than correctly specified relaxed landmarking in MSE (see Supplemental Figure 2). As this is also pronounced in the BS, we expect to notice this in real-life applications as well. Censoring degrees seem to only influence the correctly specified relaxed landmarking methods, whereas incorrectly specified never show good predictive potential and are therefore not influenced much by the censoring rates.

Overall scenarios and all landmark times, the results in BS and tdAUC align very well: whenever a method performs better/worse in tdAUC it also does so in BS. Unfortunately, the patterns we see in MSE are completely not visible in either tdAUC or BS. This is alarming as it means that in a real-life application it will be impossible to conclude whether a method is really recovering the true survival probabilities well and therefore whether it is appropriate to use for dynamic prediction purposes.

In conclusion, misspecifying the data generation mechanism can lead to very unstable estimates in the models. This can lead to poor predictive power, but sometimes the reverse can be visible in Brier or tdAUC scores. It is crucial to perform strict landmarking to obtain good predictive performance. Using a relaxed approach might seem like a good idea due to the increase in available information to build the model, but results in biased estimates and poor predictions. The degree of censoring only has an influence on relaxed landmarking models, and only at heavy degrees of censoring around 60% . Looking purely at tdAUC/BS can give a wrong picture of the actual predictive potential of the models for dynamic prediction, making it hard to compare models based on these scores.

## 4. Application

In this section, we compare the performance of all methods previously discussed to predict time to dementia (DM) in a real-life study on AD which is a degenerative brain disease and is the most common form of DM,^33^ causing patients to experience progressively worsening cognitive capabilities. A large population of healthy and afflicted individuals is currently being followed by researchers in the ongoing ADNI study (https://adni.loni.usc.edu/). Multiple genetic and longitudinal biomarkers are being collected over the duration of the study, such as structural MRI/PET scans and questionnaires assessing the neurocognitive abilities of participants. There are currently no disease-modifying treatments available to reverse the damage caused by AD, but treatments to slow the progression of the disease are available.^34^ Seeing as many countries have an aging population, estimating the remaining time in which an individual will be disease free is becoming more and more important.

### 4.1. ADNI data description

ADNI is an ongoing observational study initiated in October 2004 . One of the main goals of the study was to detect AD at an early stage and track disease progression using biomarkers. For this reason, multiple participants were followed by periodically collecting information such as MRI/PET images, genetics, cognitive tests as well as blood biomarkers. Some information is only collected at the initial admission to the study (baseline covariates) while other information is collected at each follow-up (longitudinal covariates). At the moment of analysis, the study contained information on 2428 participants. At each follow-up visit, patients are classified into one of the following categories based on their cognitive condition: cognitively normal (CN), mild cognitive impairment (MCI) or DM. Usually, patients progress from CN to MCI and finally to DM, but sometimes MCI is not observed, allowing for a direct transition from CN to DM.

Our goal is to predict time to DM for individuals that enter the study as MCI or CN. Therefore we restrict our attention to participants without DM at baseline. As a result, we exclude 374 participants diagnosed with DM at baseline. Our interest lies specifically in the prediction of survival probabilities using longitudinal measurements, hence we exclude 105 participants with no follow-up diagnoses. Finally, we also exclude 65 subjects with incomplete information at baseline. Our final analyses are performed using the retained 1643 participants. For the individuals that passed away before developing DM, we consider their outcome to be right-censored. We considered the baseline covariates gender, age, years of education, diagnosis at baseline and number of Apolipoprotein ϵ4 alleles.^35^ A summmary of the data can be found in Table 1. We considered longitudinal variables where at least 90 percent of participants had an observed value over the total follow-up duration, retaining 21 of the 41 available variables.

**Table 1.** Description of the ADNI data set.

ADNI Data ( n=1625 participants)		
Discrete variables	Percent	Count
Gender		
Female	46.4%	754
Male	53.6%	871
Baseline status		
CN	43%	700
MCI	57%	925
Apolipoprotein ϵ4		
0	58.2%	945
1	34.3%	558
2	7.5%	122
Final diagnosis		
AD	24.6%	400
Censored	75.4%	1225
Continuous variables	**Median (IQR)**	**Range**
Age (Years)	73.1 (68.3 - 78)	55 - 91.4
Education (Years)	16 (14 - 18)	4 - 20
Event time distribution (Years)		
AD	2.02 (1.03 - 4)	0.45 - 13.05
Censored	3.38 (2.03 - 6.8)	0.39 - 15.73

ADNI: Alzheimer Disease Neuroimaging Initiative; MCI: mild cognitive impairment; CN: cognitively normal; AD: Alzheimer’s disease; IQR: interquartile range.

Study participants were followed up over half-year intervals for at most 15.5 years, with an extra follow-up a quarter year after their initial assessment (at most 32 follow-up times). Most participants failed to show on the largest part of planned assessments, and many variables were not recorded at every follow-up. The retained 21 variables have an extremely large proportion of missing values. On average over all considered variables, patients have a median of 3.7 (IQR 2−5.37 ) out of the possible 33 values recorded. Over the 21 variables, the median percentage of recorded values is only 13.1% (IQR 11.8−13.2 ). We are therefore working in a very sparse setting when considering time-on-study, and even more so when considering the age-at-observation setting.

### 4.2. Results

As discussed in Section 2.3.4 the strictly landmarked model is built on less data than the relaxed model. Due to the low density of observations at early time points in some variables, we cannot fit the strictly landmarked model on all 21 considered variables using landmark times before 3.5 years. For example, a landmark time of 3 years would require us to consider only 20 of the considered variables. For consistency we therefore consider landmark times at 3.5,4,4.5 and 5 years after the start of the study, attempting to predict survival probabilities for participants still event free at these landmark times. We study the effect of the different landmarking methods, age-based centering and penalization (see Section 2.6) on predictive performance. We use MFPCCox^8^ as reference method, which employs relaxed landmarking, no age-based centering and no penalization. Predictive performance is evaluated at half-year intervals after landmark time by means of time dependent AUC and BS employing repeated cross-validation with 5 folds and 20 repetitions. The parameter M was chosen so that ${\mathrm{PVE}}^{\left(q\right)}>0.93$ for all q=1,…,Q .

**Figure 5.**
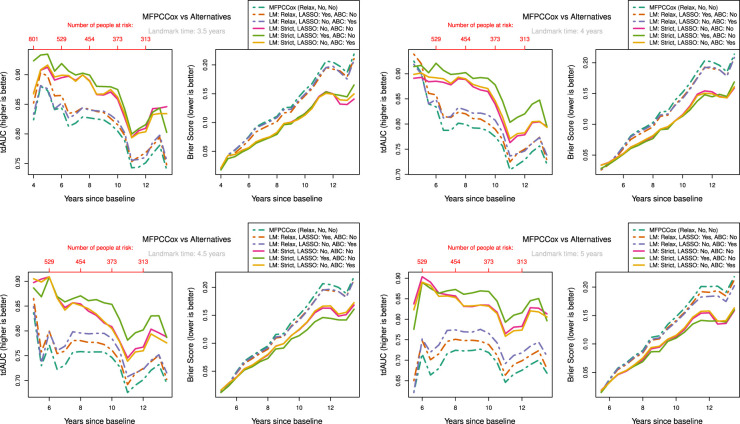
Measure of performance for LM, LASSO regularization (LASSO) and ABC methods at different landmark times on ADNI data. Validation scores were determined by using 20 times repeated 5 -fold cross validation. Dashed lines: Relaxed landmarked methods. Solid lines: Strict landmarked methods. MFPCCox (LM: Relax, LASSO: No, ABC:No)^8^ used as reference method. (a) Landmark time: 3.5 years; (b) Landmark time: 4 years; (c) Landmark time: 4.5 years; (d) Landmark time: 5 years. LM: landmark; ABC: age-based centered; ADNI: Alzheimer Disease Neuroimaging Initiative.

The results are shown in Figure 5. We can see a clear distinction in performance between strict landmarking and relaxed landmarking methods, with strict landmarking performing better on both tdAUC and BS. The overall best-performing method seems to be the LASSO regularized strictly landmarked MFPCCox (lightgreen line). MFPCCox has the worst predictive performance over all landmark times, with any considered addition improving on this. Over all landmark times, we can see that methods performing better in tdAUC also perform better in BS and vice versa. We also observed this phenomenon in our simulation study in Section 3. Additionally, the best-performing relaxed landmarking method changes from regularized MFPCCox at early landmark times to age-based centered MFPCCox at later landmark times. The age-based centered and uncentered strictly landmarked methods have near identical performance, at all times outperforming the best relaxed landmarking method.

Interestingly, for strictly landmarked models age-based centering does not seem to improve prediction accuracy at all, while for relaxed landmarked models age-based centering improves prediction as the landmark time becomes larger (see Figure 5(c) & (d)). A possible reason for this is that with strict landmarking the mean on the age time scale $\mu (t,a_{i})$ is very hard to estimate, especially in the extremely sparse setting we are working in. There are two causes: at early landmark times the mean function is determined using only a small time span for each patient. At late landmark times most patients will already have had the event or been censored, leaving only very little participants to determine the mean function, albeit over a longer time span. Relaxed landmarking methods do not have this problem as all training participants are considered over their entire study time, therefore allowing to use more data points to estimate the mean on age-at-observation scale.

A notable effect of using relaxed landmarked methods is the sharp drop in prediction accuracy right after the landmark time when compared to strictly landmarked methods. This happens because the relaxed model fit on the training data is not necessarily representative for participants who are event-free at landmark time and is one of the main reasons to consider strict landmarking. Finally, LASSO regularization seems to improve prediction accuracy for both strictly and relaxed landmarked methods, although not by much.

Remember that age is included as baseline predictor in all considered methods. Examining the Cox prediction models, we find that when not using ABC age is not found to be a significant predictor of DM. This is the case for both strict and relaxed landmarking methods. On the other hand, when using ABC age is found to be a significant predictor. As an example, in one of the evaluation folds we found p-values for age of 0.65/0.73 for the relaxed/strict non-ABC methods as opposed to 0.0001/0.003 for the ABC-methods. Inspecting the scores of non-ABC mFPCA we found that they correlate strongly with the age of patients. This means that in non-ABC methods the effect of age is already contained in the estimated scores. A possible downside of this is that the age effect might be masking more interesting prediction trends contained in the longitudinal data. The main benefit of ABC is that it can remove this age trend to uncover potentially more interesting predictive indicators. As age becomes a significant predictor in ABC methods, this seems to be successful. However, as we do not significantly improve prediction accuracy by using ABC, it seems that not all longitudinal variables are influenced by the age of patients.

Let us compare the results of the application with those of the simulation study in Section 3. In the ADNI data around 60% of all events were censored, meaning that we should compare with the heavy censoring scenarios. Additionally, most of the events take place in the first 3 years of the study, so we can compare with the simulation results for a landmark time at 9 years. The comparison between the analysis on the ADNI data and the simulation study would also suggest that at least some of the longitudinal covariates benefit from being considered at the age-at-observation time scale, but some may not. Additionally, we find that regularization improves the prediction accuracy, implying that not all scores extracted by mFPCA methods hold predictive power or that they are correlated with the baseline predictors.

## 5. Discussion

In this article we introduced the concept of relaxed and strict landmarking. We developed an age-based centred mFPCA procedure, which can be used to remove the variation due to the difference in age at baseline of subjects participating in a study. Even though our methodology focused on age as centring variable, the procedure can be extended to any time-dependent variable. Finally, we used a regularized Cox model to dynamically predict time to DM.

Results based on the simulation study and on the real data application show that improperly landmarking can lead to biased results in dynamic prediction models, thereby strongly decreasing predictive accuracy. It is therefore important to use strict landmarking approaches so that accurate predictions can be made. In practice, it might not always be possible or desirable to use strict landmarking. For the ADNI data, we did not consider landmark times before 3.5 years as many longitudinal variables did not have a sufficient amount of observations before earlier landmark times to determine the mean function using thin plate spline regression. A possible solution could be to use a different method for mean smoothing; here this was not desirable as the method of smoothing was not the focus of this article. Another possibility would be to use ‘truncated relaxed’ landmarking, where instead of full information only part of follow-up is used for training the model (i.e. 3.5 years of follow-up for predictions at landmark time 2 ).

In the analysis of the ADNI data set, there is a big improvement in prediction accuracy when using age-based centring methods with relaxed landmarking. The results from our simulation study suggest that although using an age-based centred procedure might be appropriate for the ADNI data, the largest gain in predictive potential can be gained by using a strict landmarking procedure. In other words, the landmarking bias outweighs the bias incurred by incorrectly specifying the underlying data generation mechanism. A limitation is that we have considered only two options: Age-based centring for all variables or for none. Exploring all possible combinations of variables to use for age-based centring was not feasible; with proper medical knowledge it might be possible to consider only few covariates for age-based centring. It is therefore possible that an improvement in prediction accuracy can be achieved when considering only a relevant subset of covariates. This is especially emphasized by the result from the simulation study which showed that using a time-on-study model for age-at-observation data resulted in very poor predictive accuracy.

In the ADNI study, all subjects did not follow the study plan, either by not showing up to the planned follow-ups or at the planned dates. As a consequence the resulting longitudinal information is sparse and the observed time grid is not regular. Even though FPCA methods do not strictly require a regular grid, it becomes computationally infeasible to estimate the mean and covariance functions on an irregular grid when observations are very sparse. We therefore assumed the grid to be regular by considering the follow-up to have happened at the closest planned assessment time. This can have a great effect on the estimation procedures, affecting the resulting scores and eigenfunctions such that they do not represent subject progressions appropriately. On the other hand, mixed modelling approaches such as pencal^11^ do not require a regular grid, even in a sparse setting, but do require an assumption on the functional form of the underlying linear structure for the longitudinal covariates. Besides this, it is easier to perform strict landmarking as no smoothing of the mean/covariance functions is required. These approaches can therefore be better suited for sparse data.

JM approaches are often employed to model survival and longitudinal outcomes jointly. Li et al.^8^ have performed a simulation study to compare the performance of MFPCCox with that of a JM approach. They found that when the parametric form of the joint model was misspecified, MFPCCox achieved comparable predictive performance while more accurately recovering the underlying longitudinal trajectories. As our proposed methodology has been shown to improve on MFPCCox, we expect to outperform JM approaches even further. Li et al.^8^ also discuss the computational burden of JM approaches, noting that the JM approaches are not computationally feasible with more than six longitudinal variables. We can therefore not fit a joint model on the ADNI data considered in Section 4.

A different approach for the multivariate principal component analysis decomposition (mFACEs), using tensor product B-splines to estimate the covariance function directly was proposed in Li et al.^16^ The authors show in a simulation study in a sparse setting that mFACEs captures cross-correlation between functions and recovers the eigenfunctions and eigenvalues better than mFPCA, as well as better predicting the longitudinal biomarkers in the ADNI data. As mFACEs uses a multivariate version of PACE (3) for score estimation, this could mitigate the problem of missing data discussed in Section 2.3.4, possibly allowing for earlier predictions with strict landmarking. Additionally, age-based centring can also be incorporated into the mFACEs procedure. Lin et al.^9^ showed that mFPCA performed slightly better in survival prediction than mFACEs when using relaxed landmarking with a Cox model, both in a simulation study as well as on the ADNI data. They also concluded that RSFs outperformed Cox models for survival prediction. These results should be re-evaluated with a strict landmarking approach, as the bias incurred by relaxed landmarking might favour RSF over Cox models.

## Supplemental Material

sj-pdf-1-smm-10.1177_09622802231224631 - Supplemental material for Dynamic prediction of survival using multivariate functional principal component analysis: A strict landmarking approach Supplemental material, sj-pdf-1-smm-10.1177_09622802231224631 for Dynamic prediction of survival using multivariate functional principal component analysis: A strict landmarking approach by Daniel Gomon, Hein Putter, Marta Fiocco and Mirko Signorelli in Statistical Methods in Medical Research
