# Supplementary material for: Dynamic prediction of survival using multivariate functional principal component analysis: A strict landmarking approach
Source: Stat Methods Med Res. 2024 Jan 9;33(2):256–72. doi: 10.1177/09622802231224631 (PMC10928955; doi:10.1177/09622802231224631)
Supplement: sj-pdf-1-smm-10.1177_09622802231224631 - Supplemental material for Dynamic prediction of survival using multivariate functional principal component analysis: A strict landmarking approach [file sj-pdf-1-smm-10.1177_09622802231224631.pdf]

Journal Title
XX(X):1–2
©The Author(s) 2016
Reprints and permission:
sagepub.co.uk/journalsPermissions.nav
DOI: 10.1177/ToBeAssigned
www.sagepub.com/
SAGE

Supplemental material for Dynamic prediction of survival using multivariate Functional Principal Component Analysis: a strict landmarking approach

Daniel Gomon[1], Hein Putter [2], Marta Fiocco[1, 2] and Mirko Signorelli[1]

Keywords
Dynamic prediction, Landmarking, Survival, Functional principal component analysis

[1]Mathematical Institute, Leiden University, Leiden, the Netherlands
[2]Department of Biomedical Data Sciences, Leiden University Medical Centre, Leiden, the Netherlands

Corresponding author:
Daniel Gomon, Mathematical Institute, Niels Bohrweg 1, 2333CA Leiden, the Netherlands
Email: d.gomon@math.leidenuniv.nl

A. Simulation study - additional censoring scenarios

In this Section we display some additional simulation results that highlight the effect of different censoring scenarios on the performance of the models. The scenarios shown here are:

- Scenario 1: Time-on-study data and light censoring ($\approx 20\%$ of observations censored).
- Scenario 3: Time-on-study data and heavy censoring ($\approx 60\%$ of observations censored).
- Scenario 4: Age-at-observation data and light censoring ($\approx 20\%$ of observations censored).
- Scenario 6: Age-at-observation data and heavy censoring ($\approx 60\%$ of observations censored).

The underlying multivariate data and survival times are generated identically to the procedure described in the article. Right-censoring times C_i were generated from an exponential distribution with rate $\exp(-3.5)$ (light), $\exp(-2.75)$ (median), $\exp(-2)$ (heavy) to obtain the censoring percentages stated above.

The figures below display the performance measures of the considered methods for the different scenarios.

References

1. Li K and Luo S. Dynamic prediction of Alzheimer's disease progression using features of multiple longitudinal outcomes and time-to-event data. *Stat Med* 2019; 38: 4804–4818.

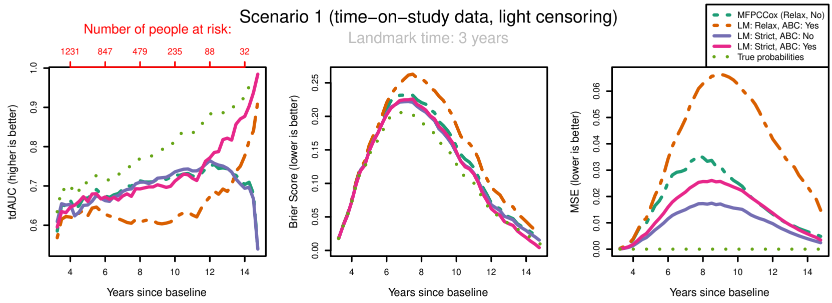

(a) Landmark time: 3 years

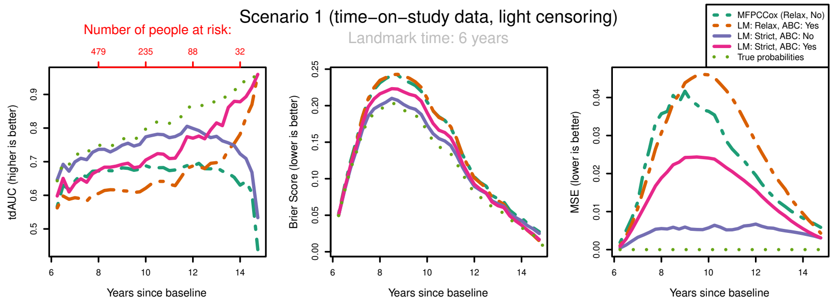

(b) Landmark time: 6 years

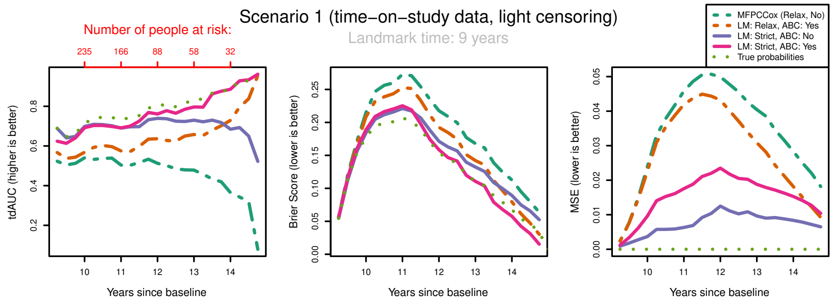

(c) Landmark time: 9 years

Figure 1. Time dependent AUC (tdAUC), Brier Score and MSE in the third scenario (time-on-study data, light censoring) for the considered methods over landmark times at $3, 6$ and 9 years after baseline. Landmark method ("LM"); age-based centered ("ABC"). Dashed lines: relaxed landmarked methods. Solid lines: strictly landmarked methods. Dotted lines: true probabilities. MFPCCox [1] (LM: Relax, LASSO: No, ABC:No) used as reference method. Number of people at risk at evaluation times displayed in red.

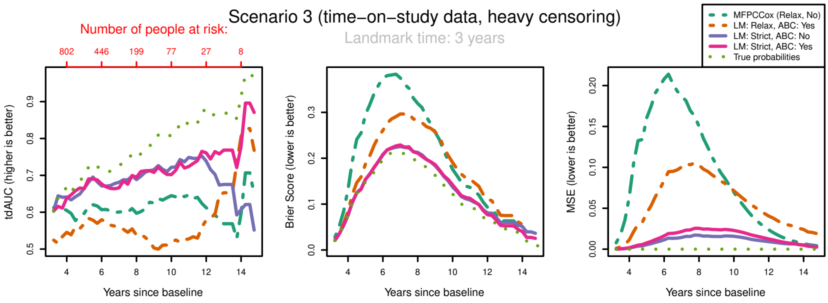

(a) Landmark time: 3 years

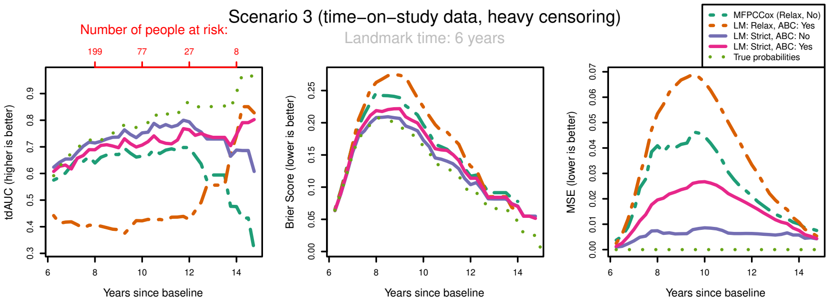

(b) Landmark time: 6 years

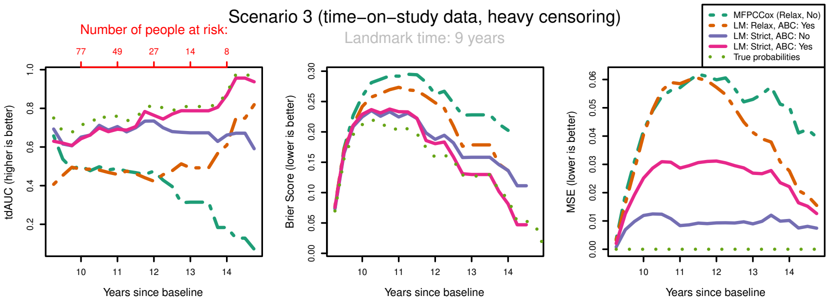

(c) Landmark time: 9 years

Figure 2. Time dependent AUC (tdAUC), Brier Score and MSE in the fifth scenario (time-on-study data, heavy censoring) for the considered methods over landmark times at $3, 6$ and 9 years after baseline. Landmark method ("LM"); age-based centered ("ABC"). Dashed lines: relaxed landmarked methods. Solid lines: strictly landmarked methods. Dotted lines: true probabilities. MFPCCox[1] (LM: Relax, LASSO: No, ABC:No) used as reference method. Number of people at risk at evaluation times displayed in red.

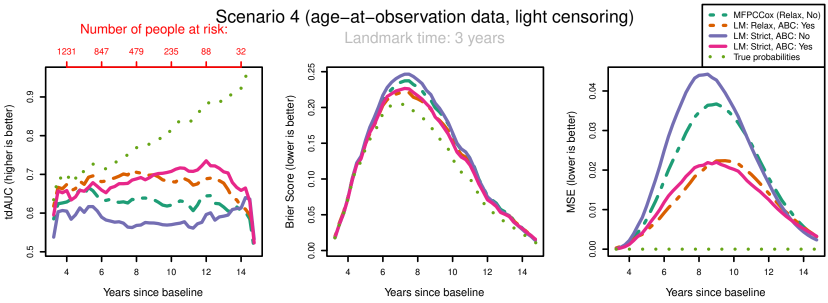

(a) Landmark time: 3 years

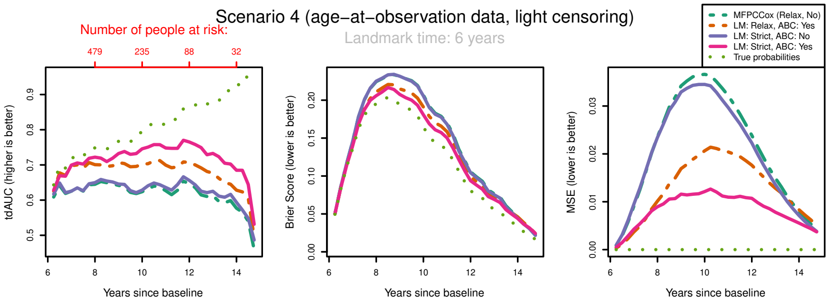

(b) Landmark time: 6 years

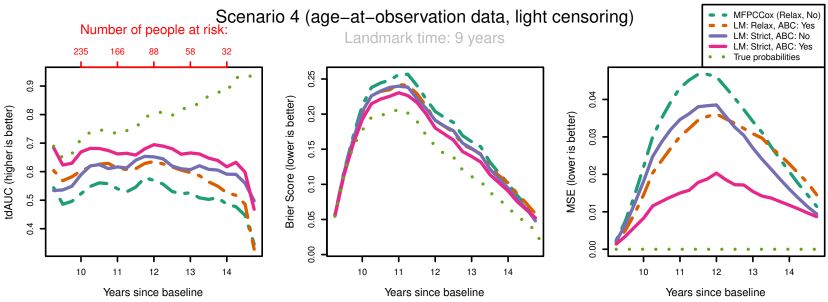

(c) Landmark time: 9 years

Figure 3. Time dependent AUC (tdAUC), Brier Score and MSE in the sixth scenario (age-at-observation data, light censoring) for the considered methods over landmark times at $3, 6$ and 9 years after baseline. Landmark method (“LM”); age-based centered (“ABC”). Dashed lines: relaxed landmarked methods. Solid lines: strictly landmarked methods. Dotted lines: true probabilities. MFPCCox [1] (LM: Relax, LASSO: No, ABC:No) used as reference method. Number of people at risk at evaluation times displayed in red.

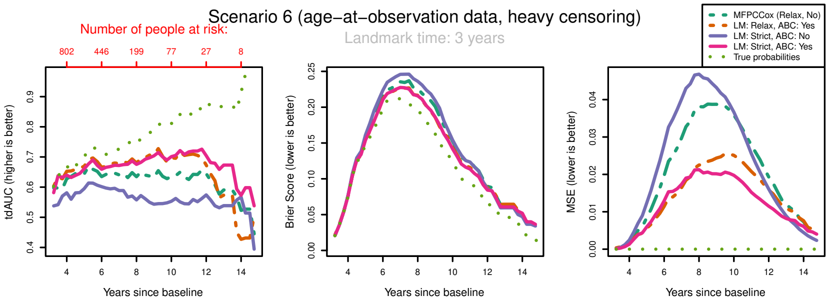

(a) Landmark time: 3 years

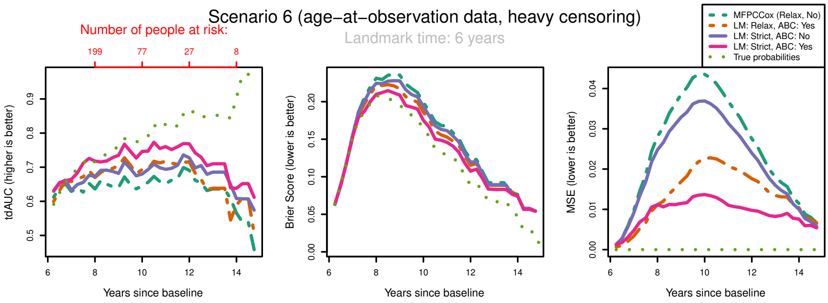

(b) Landmark time: 6 years

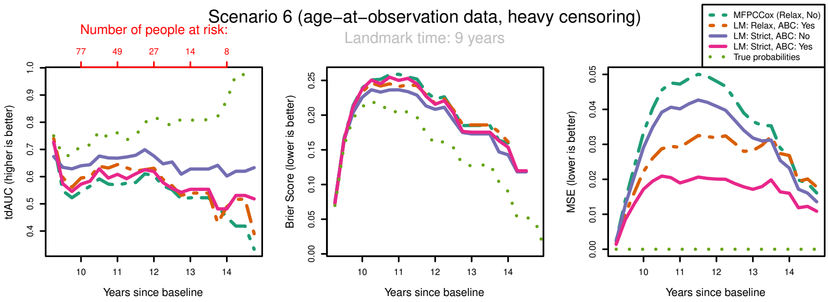

(c) Landmark time: 9 years

Figure 4. Time dependent AUC (tdAUC), Brier Score and MSE in the eight scenario (age-at-observation data, heavy censoring) for the considered methods over landmark times at $3, 6$ and 9 years after baseline. Landmark method (“LM”); age-based centered (“ABC”). Dashed lines: relaxed landmarked methods. Solid lines: strictly landmarked methods. Dotted lines: true probabilities. MFPCCox[1] (LM: Relax, LASSO: No, ABC:No) used as reference method. Number of people at risk at evaluation times displayed in red.
